# Changes in survival over time for primary brain and other CNS tumors in the United States, 2004–2017

**DOI:** 10.1007/s11060-022-04138-w

**Published:** 2022-10-05

**Authors:** Gino Cioffi, Kristin A. Waite, Jacob L. Edelson, Carol Kruchko, Quinn T. Ostrom, Jill S. Barnholtz-Sloan

**Affiliations:** 1 Trans Divisional Research Program, Division of Cancer Epidemiology and Genetics, National Cancer Institute, Bethesda, MD USA; 2 Central Brain Tumor Registry of the United States, Hinsdale, IL USA; 3 Department of Neurosurgery, Duke University School of Medicine, Durham, NC USA; 4 The Preston Robert Tisch Brain Tumor Center, Duke University School of Medicine, Durham, NC USA; 5 Duke Cancer Institute, Duke University Medical Center, Durham, NC USA; 6 Center for Biomedical Informatics & Information Technology, National Cancer Institute, Bethesda, MD USA; 7 National Cancer Institute, Shady Grove Campus 9609 Medical Center Dr, Rockville, MD 20850 USA

**Keywords:** Brain tumors, Central nervous system tumors, Epidemiology, Survival

## Abstract

**Purpose:**

Despite advances in cancer diagnosis and clinical care, survival for many primary brain and other central nervous system (CNS) tumors remain poor. This study performs a comprehensive survival analysis on these tumors.

**Methods:**

Survival differences were determined utilizing the National Program of Cancer Registries Survival Analytic file for primary brain and CNS tumors. Overall survival and survival of the 5 most common histopathologies, within specific age groups, were determined. Overall survival was compared for three time periods: 2004–2007, 2008–2012, and 2013–2017. Survival differences were evaluated using Kaplan–Meier and multivariable Cox proportional hazards models. Models were adjusted for sex, race/ethnicity, and treatment. Malignant and non-malignant brain tumors were assessed separately.

**Results:**

Among malignant brain and CNS tumor patients overall, there were notable differences in survival by time period among all age groups. Similar differences were noted in non-malignant brain and CNS tumor patients, except for adults (aged 40–64 years), where no survival changes were observed. Survival differences varied within specific histopathologies across age groups. There were improvements in survival in 2008–2012 and 2013–2017, when compared to 2004–2007, in children, AYA, and older adults with malignant tumors, and among older adults with non-malignant tumors.

**Conclusion:**

Overall survival for malignant brain and other CNS tumors improved slightly in 2013–2017 for all age groups as compared to 2004–2007. Significant changes were observed for non-malignant brain and other CNS tumors among older adults. Information regarding survival over time can be utilized to identify population level effects of diagnostic and treatment improvements.

**Supplementary Information:**

The online version contains supplementary material available at 10.1007/s11060-022-04138-w.

## Introduction

In 2018, approximately 88,190 new cases of primary malignant and non-malignant brain and other central nervous system (CNS) tumors were diagnosed in the United States (US), with non-malignant tumors occurring more than twice as frequently as malignant tumors [1, 2]. From 2001 to 2017, following diagnosis of a primary malignant brain or other CNS tumor, the 5-year relative survival rate was 66.9% [1]. During this same time period, 5-year relative survival rate, following diagnosis, was longer for individuals aged 0–14 years and 15–39 years compared to individuals who are age 40 years and older, with 5-year relative survival rates being 84.1%, 84.7%, and 65.6% respectively [1]. For primary non-malignant brain and other CNS tumors the 5-year relative survival rate was 92.1%. Individuals aged 15–39 years and individuals aged 0–14 years have the highest 5-year relative survival rates compared to individuals who were age 40 years and older; 98.3%, 97.5%, and 90.7%, respectively [1].

Factors such as age at diagnosis, sex, race/ethnicity, and treatment pattern can have significant effects on the survival of primary brain and other CNS tumors. In most cases, females diagnosed with primary brain and other CNS tumors have better survival outcomes when compared to males, while individuals who are Black, non-Hispanic generally have a poorer survival outcome when compared to non-Hispanic White individuals. Tumor site and histopathology significantly affect survival. In general, individuals aged 15–39 years, considered adolescent and young adults (AYA), have a better overall 5-year survival when compared to children (age 0–14 years) and adults over age 40 years among both malignant and non-malignant tumors [1]. Adults, 40 years or older diagnosed with a primary malignant brain and other CNS tumor have the worst survival rates in almost every histopathologic subtype.

These survival differences may be impacted by histopathology distribution, which varies by age group, as tumor site and histopathology significantly affect survival. Tumors that occur in the acoustic nerves have the highest 5-year survival rate (99.5%) while tumors of the parietal lobe and those of overlapping lesion of the brain have the lowest survival rate (25.2%), across all behaviors. With regards to histopathology, there is a wide range of 5-year relative survival rates with pilocytic astrocytoma having the best prognosis, and glioblastoma having the worst, 94.7% and 6.8%, respectively.

The initial treatment for brain and other CNS tumors is surgery, and the extent of surgical resection (EOR) has been shown to be a positive prognostic factor for malignant brain tumors [3, 4]. In addition, radiation and chemotherapy are regularly used together to treat primary brain and other CNS tumors. Some treatment improvements have occurred, such as In 2005, when Stupp and coworkers found that when compared to treatment with either only radiation or only temozolomide, radiation with concomitant temozolomide treatment increased survival for individuals with glioblastoma by 2.5 months [5]. Despite the increase in survival for glioblastoma, for many types of primary brain and other CNS tumors the optimal treatment remains elusive.

Survival over time of primary brain and other CNS tumors have not been analyzed in a rigorous, comprehensive manner that includes both malignant and non-malignant tumors. This study evaluates survival over time for primary brain and other CNS tumors, from 2004 to 2017, using the most complete and up-to-date overall survival data from the Center for Disease Control’s (CDC) National Program of Cancer Registries (NPCR) and assessing the five most common histopathologies by age group.

## Methods

This study was conducted under Institutional Review Board approval from Duke University School of Medicine.

### Data collection

De-identified survival data, containing primary, first sequence brain and other CNS tumor information from 2004 to 2017 with either histopathologic or radiographic confirmation, were obtained from the CDC’s NPCR survival database, which consisted of 42 NPCR registries containing population-based information for 82% of the U.S. population. Behavior status was identified using *The International Classification of Disease for Oncology, Third Edition* (ICD-O-3) behavior codes (0,1: non-malignant; 3: malignant), and specific histopathologies were classified according to the 2021 Central Brain Tumor Registry of the United States (CBTRUS) Annual Report [1, 6, 7]. In a departure from CBTRUS normal reporting, pilocytic astrocytoma was classified as non-malignant due to the clinical behavior designation of these tumors [8].

### Statistical analysis

For this analysis year of diagnosis was divided into 3 time periods to analyze survival over time. Time periods of study were divided into periods of roughly the same time length for year of diagnosis: 2004–2007, 2008–2012, and 2013–2017. Individuals were divided into the following age groups, based upon age of diagnosis: children 0–14 years at diagnosis, adolescents, and young adults [AYA] [9] 15–39 years at diagnosis, adults 40–64 years at diagnosis, and older adults 65 + years at diagnosis. Race/ethnicity was defined as Non-Hispanic/White, Non-Hispanic/Black, Non-Hispanic/Other (including Alaskan Native, American Indian, Asian, and Pacific Islander), or Hispanic/All Races. Treatment patterns were defined based upon radiation and surgery received, with cases defined as either surgery only, radiation only, or surgery and radiation. Surgery was defined by SEER site specific surgery codes for primary brain and CNS tumors using the following codes: no surgery (00) and surgery (20,21,30,40,55). Radiation was defined either as radiation received (had RAD) or radiation not received (No RAD or RAD refused). Treatment was defined as unknown if either surgery or radiation status was unknown. Descriptive statistics were assessed for age at diagnosis, sex, race/ethnicity, surgery, and radiation for each time-period using SEER*Stat (version 8.3.9.2). The five most common histopathologies were also assessed by age group, as histopathology distribution varies by age. Histopathologies were classified as malignant or non-malignant based upon behaviors and as reported in the *Central Brain Tumor Registry of the United States* annual report which are organized by clinically relevant histopathology groupings reflecting the *2016 World Health Organization (WHO) Classification of Tumours of the Central Nervous System* [10]. Tumors with ICD-O-3 behavior codes of /0 for benign and /1 for uncertain were classified as non-malignant while those with ICD-O-3 behavior codes of /3 were classified as malignant. Historical language and histopathological nomenclature at the time of diagnosis is utilized for this analysis. Kaplan Meier analysis was performed to assess differences in overall survival by time-period by these age groups. Kaplan Meier survival curves were generated for the five most common histopathological groups found within each age group. Histopathology-specific analyses for non-malignant tumors among individuals ages 0–14years were excluded due to the low number of events. Survival cannot be estimated when there are less than 50 cases or 16 deaths per the CBTRUS agreement with CDC. Log rank tests were performed to evaluate differences in survival curves. Age-stratified multivariable Cox proportional hazard models assessing overall survival for time period, adjusted for sex, race/ethnicity, and treatment pattern, were performed and hazard ratios (HR) and associated 95% confidence intervals (CI) are reported. The Cox proportional hazard assumptions were tested using Schoenfeld residuals and were not found in violation.

P-values less than 0.05 were considered statistically significant for all analyses. All tables and figures were generated using R Software (version 4.1.0).

## Results

### Descriptive statistics

Between 2004 and 2017, there were 841,430 primary, brain and other CNS tumors diagnosed, of which 225,685 (27%) were malignant and 615,745 were (73%) non-malignant (Table 1). The overall male to female ratio was consistent over time, with a ratio of 1.3:1 (male:female) in individuals diagnosed with malignant tumors and a ratio of 1:1.8 (male:female) in individuals diagnosed with non-malignant tumors. Over the three time periods, (2004–2007, 2008–2012, 2013–2017), the number of cases diagnosed increased. This increase over time was observed in all age groups for both malignant and non-malignant tumors. The distribution of individuals diagnosed remained relatively consistent over time with regards to race/ethnicity (Table 1). For patients with non-malignant tumors, the distribution of individuals receiving only surgery decreased from 42% in 2004–2007 to 29% in 2013–2017.

**Table 1:** Characteristics of primary brain and other CNS tumors stratified by behavior and time period of diagnosis (NPCR Survival Data: Data Provided by CDC's National Program of Cancer Registries SEER*Stat Database: NPCR Survival Analytic file, 2004-2017)*

Characteristic	Malignant^a^			Non-malignant		
	2004–2007 N = 61,391 n(%)	2008–2012 N = 79,918 n(%)	2013–2017 N = 84,376 n(%)	2004–2007 N = 140,038 n(%)	2008–2012 N = 220,879 n(%)	2013–2017 N = 254,828 n(%)
Age group						
0–14	5,189 (8.5%)	6,739 (8.4%)	7,009 (8.3%)	5,008 (3.6%)	7,270 (3.3%)	8,026 (3.1%)
15–39	9,587 (16%)	12,125 (15%)	12,247 (15%)	22,662 (16%)	34,500 (16%)	37,866 (15%)
40–64	25,641 (42%)	33,611 (42%)	33,990 (40%)	60,517 (43%)	94,140 (43%)	104,220 (41%)
65+	20,974 (34%)	27,443 (34%)	31,130 (37%)	51,851 (37%)	84,969 (38%)	104,716 (41%)
Sex						
Female	27,314 (44%)	35,292 (44%)	37,069 (44%)	90,460 (65%)	142,435 (64%)	163,108 (64%)
Male	34,077 (56%)	44,626 (56%)	47,307 (56%)	49,578 (35%)	78,444 (36%)	91,720 (36%)
Race/ethnicity						
Non-Hispanic White	48,913 (80%)	62,268 (78%)	64,272 (76%)	102,140 (73%)	156,277 (71%)	175,158 (69%)
Non-Hispanic Black	4,380 (7.1%)	5,930 (7.4%)	6,279 (7.4%)	16,193 (12%)	27,827 (13%)	33,051 (13%)
Non-Hispanic Other	1,884 (3.1%)	2,986 (3.7%)	3,552 (4.2%)	5,627 (4.0%)	9,672 (4.4%)	13,052 (5.1%)
Hispanic (all races)	5,921 (9.6%)	8,314 (10%)	9,739 (12%)	14,617 (10%)	24,898 (11%)	30,818 (12%)
Unknown	293 (0.5%)	420 (0.5%)	534 (0.6%)	1,461 (1.0%)	2,205 (1.0%)	2,749 (1.1%)
Treatment pattern						
No treatment	9,672 (16%)	12,081 (15%)	11,788 (14%)	58,098 (41%)	111,536 (50%)	139,009 (55%)
Radiation only	7,942 (13%)	9,566 (12%)	8,873 (11%)	8,329 (5.9%)	11,238 (5.1%)	10,474 (4.1%)
Surgery and radiation	23,591 (38%)	32,988 (41%)	37,402 (44%)	3,946 (2.8%)	5,299 (2.4%)	5,809 (2.3%)
Surgery only	12,136 (20%)	15,028 (19%)	14,190 (17%)	58,582 (42%)	74,500 (34%)	73,505 (29%)
Unknown	8,050 (13%)	10,255 (13%)	12,123 (14%)	11,083 (7.9%)	18,306 (8.3%)	26,031 (10%)

*Differences in distribution are significant but not reported due to lack of clinical relevance

^a^Individuals were classified as having malignant or non-malignant tumors based on the categories present in Table 2 of the *CBTRUS Statistical Report* [23]

### Kaplan–Meier overall survival

**Fig. 1:**
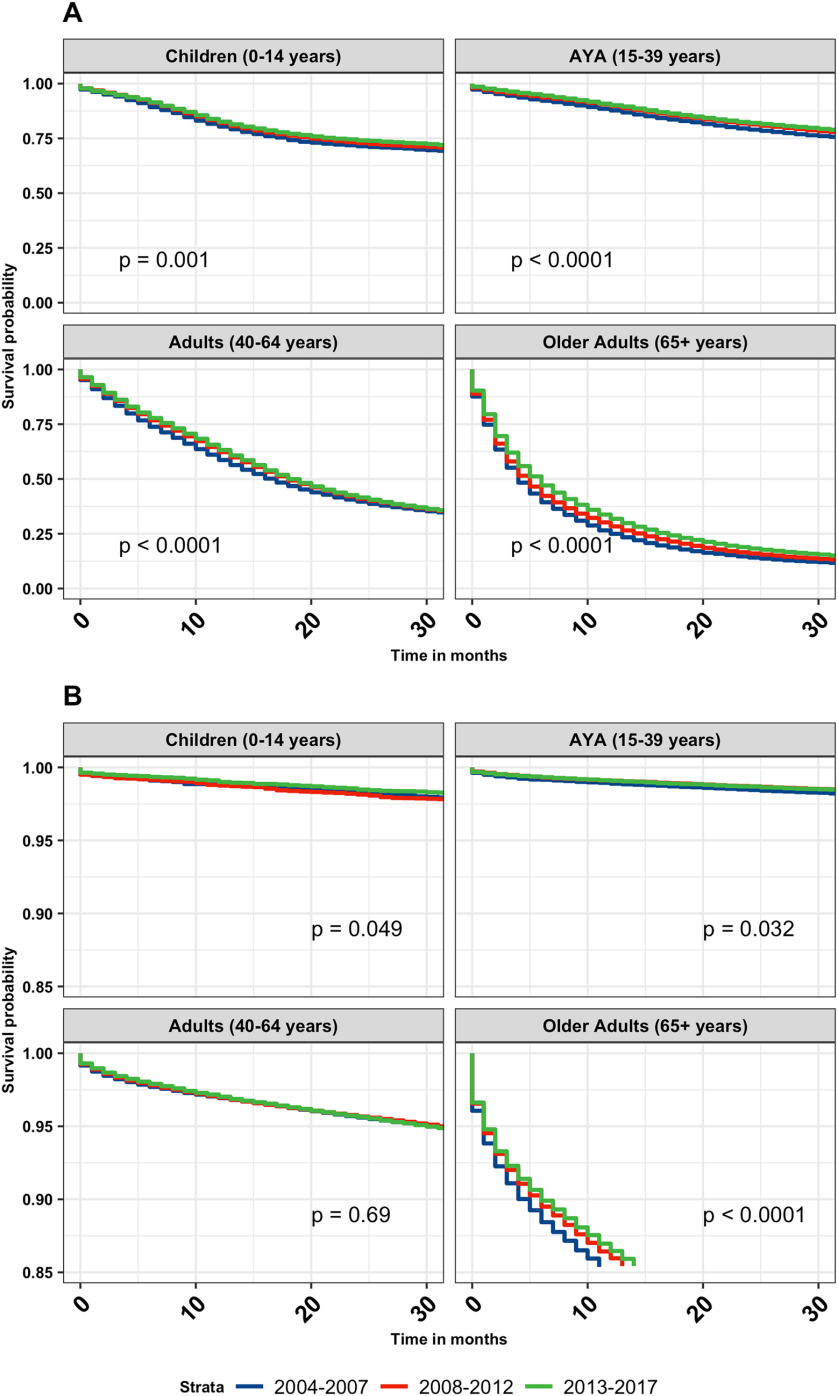
Overall Kaplan-Meier survival curves stratified by age group (0–14 years, 15–39 years, 40–64 years, 65 + years) and time period of diagnosis (2004–2007, 2008–2012, 2013–2017) for all primary brain and other CNS tumors by malignant (**A**) and non-malignant (**B**) behavior. p-values were determined by a log-rank test. (NPCR Survival Data: Data provided by CDC’s National Program of Cancer Registries SEER*Stat Database: NPCR Survival Analytic file, 2004–2017)

**Fig. 2:**
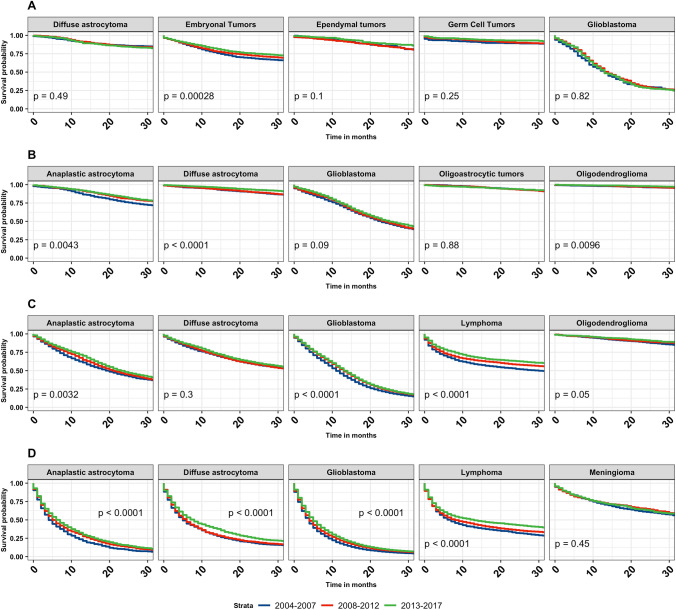
Kaplan-Meier overall survival curves for malignant brain and other CNS tumors stratified by year of diagnosis for the five most common histopathologies stratified by age group [children ages 0–14 years (**A**), AYA ages 15–39 years (**B**), adults ages 40–64 years (**C**), older adults ages 65 + years (**D**)] and time period of diagnosis (2004–2007, 2008–2012, 2013–2017). p-values were determined by a log-rank test. (NPCR Survival Data: Data provided by CDC’s National Program of Cancer Registries SEER*Stat Database: NPCR Survival Analytic file, 2004–2017)

**Fig. 3:**
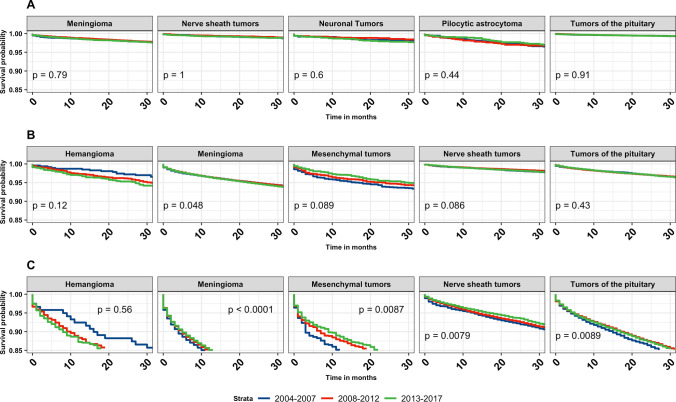
Kaplan–Meier overall survival curves for non-malignant brain and other CNS tumors stratified by year of diagnosis for the five most common pathologies stratified by age group [AYA ages 15–39 years (**A**), older adults ages 40 + years (**B**), older adults ages 65 + years (**C**)] and time period of diagnosis (2004–2007, 2008–2012, 2013–2017). p-values are determined by a log-rank test. (NPCR Survival Data: Data provided by CDC’s National Program of Cancer Registries SEER*Stat Database: NPCR Survival Analytic file, 2004–2017)

Kaplan–Meier overall survival curves for malignant and non-malignant tumors by time-period (2004–2007, 2008–2012, 2013–2017) were generated and stratified according to age group (Fig. 1). In all age groups, survival improved for individuals diagnosed with primary malignant brain and other CNS tumors (children p = 0.001, AYA p < 0.001, adults p < 0.001, and older adults p < 0.001). In individuals diagnosed with non-malignant tumors, statistically significant survival differences were noted for children (p = 0.049), AYA (p = 0.032) and older adults (p < 0.001). Increased survival was observed in children for embryonal tumors (p < 0.001). In AYA, there was an increase in survival in individuals diagnosed with anaplastic astrocytoma (p = 0.004) (Note: anaplastic is no longer used per 2021 WHO criteria, but is used here due to historical practices), diffuse astrocytoma (p < 0.001), and oligodendroglioma (p = 0.010). In adults, anaplastic astrocytoma (p = 0.003), glioblastoma (p < 0.001), and lymphoma (p < 0.001) showed statistically significant improvements in survival over time (Fig. 2). The median survival for glioblastoma in adults increased by 2 months, from 11 months (2004–2007, 95% CI 11–12) to 13 months (2013–2017, 95% CI 13–14). The median survival for anaplastic astrocytoma in adults increased by 3.5 months, from 19.5 months (2004–2007, 95% CI 18–21) to 23 months (2013–2017, 95% CI 22–25). The median survival for lymphoma in adults increased by 17 months, from 30 months (2004–2007, 95% CI 25–37) to 47 months (2013–2017, 95% CI 42–55) (Table 2). Anaplastic astrocytoma, diffuse astrocytoma, glioblastoma, and lymphoma all showed significant survival improvements in older adults (p < 0.001) (Fig. 2). Median survival for anaplastic astrocytoma in older adults increased by 3 months, from 4 months (2004–2007, 95% CI 4–5) to 7 months (2013–2017, 95% CI 6–7). Median survival for diffuse astrocytoma in older adults increased by 3 months, from 5 months (2004–2007, 95% CI 5–6) to 8 months (2013–2017, 95% CI 7–9). glioblastoma median survival in adults increased by 1 months, from 4 months (2004–2007, 95% CI 4–4) to 5 months (2013–2017, 95% CI 5–5). The median survival for lymphoma in adults increased by 6 months, from 7 months (2004–2007, 95% CI 6–8) to 13 months (2013–2017, 95% CI 11–15) (Table 2). For the non-malignant brain and CNS tumors, there was a slight increase in survival for meningioma (p = 0.048) among adult patients. Among older adults, there was a significant survival improvement for meningioma (p < 0.001), mesenchymal tumors (p = 0.009), nerve sheath tumors (p = 0.008), and tumors of the pituitary (p = 0.009) (Fig. 3).

**Table 2:** Median survival table for the five most common malignant tumors in each age group by time period of diagnosis (NPCR survival data: data provided by CDC's National Program of Cancer Registries SEER*Database: NPCR survival analytic file, 2004–2017)

Age at diagnosis	Histopathology	2004–2007			2008–2012			2013–2017		
		Cases	Deaths	Median survival months (95% CI)	Cases	Deaths	Median survival months (95% CI)	Cases	Deaths	Median survival months (95% CI)
0–14	Diffuse astrocytoma	615	124	** (**–**)	686	127	** (**–**)	641	96	** (**–**)
	Embryonal tumors	1618	740	** (**–**)	1975	770	** (**–**)	1950	470	** (**–**)
	Ependymal tumors	218	95	** (143–**)	371	126	** (117–**)	427	51	** (**–**)
	Germ cell tumors	273	48	** (**–**)	389	54	** (**–**)	429	29	** (**–**)
	Glioblastoma	247	208	13 (11–15)	385	319	14 (13–16)	395	253	13 (12–16)
15–39	Anaplastic astrocytoma	884	536	87 (77–102)	1222	568	100 (89–**)	1538	280	** (**–**)
	Diffuse astrocytoma	1724	787	** (157–**)	2151	690	** (**–**)	2048	183	** (**–**)
	Glioblastoma	1570	1314	23 (21–25)	1989	1584	24 (23–25)	2303	1076	25 (23–27)
	Oligoastrocytic tumors	834	368	** (159–**)	1101	339	** (**–**)	526	72	** (**–**)
	Oligodendroglioma	1108	342	** (**–**)	1271	189	** (**–**)	1216	34	** (**–**)
40–64	Anaplastic astrocytoma	1620	1358	19.5 (18–21)	2135	1690	21 (20–23)	2346	1164	23 (22–25)
	Diffuse astrocytoma	2223	1591	38 (34–44)	2584	1681	38 (33–41)	2152	851	39 (36–46)
	Glioblastoma	14,201	13,696	11 (11–12)	19,367	18,338	13 (13–13)	20,430	14,037	13 (13–14)
	Lymphoma	1936	1377	30 (25–37)	2522	1466	47 (42–55)	2658	959	** (**–**)
	Oligodendroglioma	1349	590	** (158–**)	1505	430	** (**–**)	1447	141	** (**–**)
65+	Anaplastic astrocytoma	906	889	4 (4–5)	1159	1121	5 (5–6)	1319	1039	7 (6–7)
	Diffuse astrocytoma	1237	1187	5 (5–6)	1333	1244	6 (5–6)	1248	886	8 (7–9)
	Glioblastoma	13,099	12,970	4 (4–4)	17,875	17,571	4 (4–4)	20,675	16,920	5 (5–5)
	Lymphoma	2047	1861	7 (6–8)	2802	2263	8 (7–10)	3409	1887	13 (11–15)
	Meningioma	571	439	42 (36–53)	526	334	51 (40–61)	584	227	46 (37–**)

** Median survival not reached

In children, median survival was not reached or did not change over time. For non-malignant tumors irrespective of age, follow up times were insufficient to calculate median survival over time (Table 2).

### Cox Proportional Hazard Models of Overall Survival

**Fig. 4:**
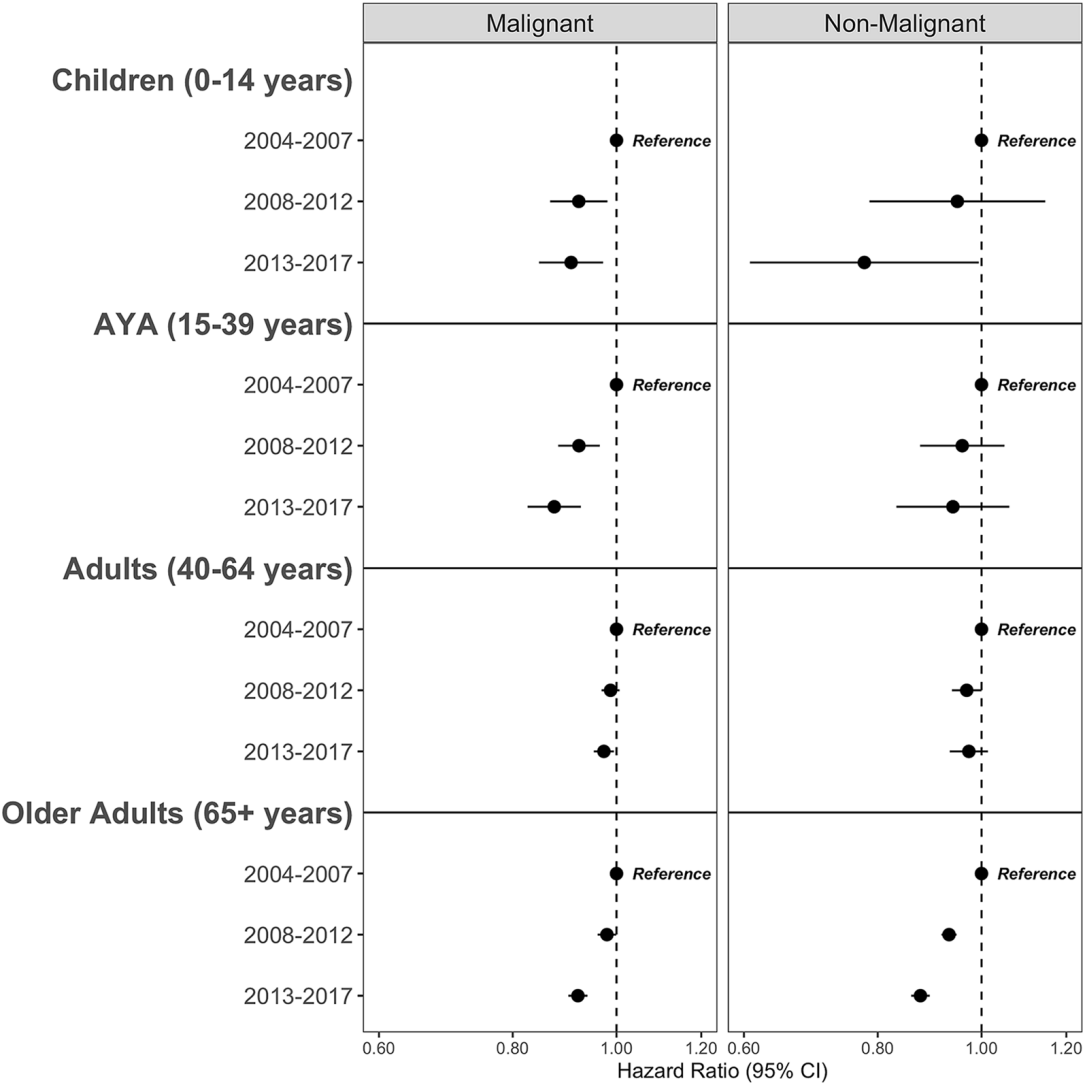
Multivariable Cox proportional hazards forest plots comparing overall survival by time period of diagnosis for primary brain and other CNS tumors stratified by behavior and age group. All models adjusted for sex, race/ethnicity, treatment pattern. (NPCR Survival Data: Data provided by CDC’s National Program of Cancer Registries SEER*Stat Database: NPCR Survival Analytic file, 2004–2017)

Cox Proportional hazards models (Fig. 4 and Supplemental Tables 1, 2) show the overall survival by time-period of diagnosis stratified by age group, and adjusted for sex, race/ethnicity, and treatment pattern (radiation and/or surgery). Overall, children, AYA, and older adults diagnosed with malignant tumors between either 2008–2012 or 2013–2017 had improvements in survival compared to individuals diagnosed earlier between 2004 and 2007 (Children: HR 0.92, 95% CI 0.87–0.98, p = 0.010 in 2008–2012; HR 0.91, 95% CI 0.85–0.97, p = 0.005 in 2013–2017; AYA: HR 0.92, 95% CI 0.88–0.96, p < 0.001, in 2008–2012; HR 0.87, 95% CI 0.83–0.93, p < 0.001 in 2013–2017; Older adults: HR 0.98, 95% CI 0.96–1.00, p = 0.043, in 2008–2012; HR 0.92, 95% CI 0.90–0.94, p < 0.001 in 2013–2017). In adults, there was a significant improvement in survival for patients diagnosed in 2013–2017 (HR 0.97, 95% CI 0.95–0.99, p = 0.014), but not for patients diagnosed in 2008–2012 (HR 0.99, 95% CI 0.97–1.01, p = 0.200 in 2013–2017). In older adults diagnosed with non-malignant tumors, there were observed improvements over time, from an earlier diagnosis (2003–2007) to a later diagnosis (HR 0.93, 95% CI 0.92–0.95, p < 0.001 in 2008–2012; HR 0.88, 95% CI 0.86, 0.89, p < 0.001 in 2013–2017).

## Discussion

This study provides an up to date and comprehensive analysis of survival trends for primary malignant and non-malignant brain and other CNS tumors in the United States. A modest, but statistically significant improvement in survival was observed for individuals diagnosed with primary malignant brain and other tumors diagnosed in the time periods 2008–2012 and 2013–2017 compared to those individuals diagnosed in between 2004 and 2007. AYA diagnosed with anaplastic astrocytoma had the largest improvement in survival with an overall 13-month survival from 2004 to 2007 to 2008–2012. Older adults with CNS lymphoma had an improvement in survival time with an overall 6-month improvement in survival from the first to last time period. Small incremental improvements were observed for individuals with other histopathologic subtypes. Among non-malignant brain and other CNS cases, there were improvements in survival for older adult patients with meningioma, mesenchymal tumors, nerve sheath tumors, and tumors of the pituitary. Due to the long survival for individuals with non-malignant brain tumors, there was likely insufficient follow-up time necessary to capture any meaningful changes in the child, AYA, and adult (40–64 years) populations.

This study presents all survival statistics stratified by age group, as the distribution of histopathology type and behavior varies greatly across ages. For example, histopathologies such as pilocytic astrocytoma, medulloblastoma, and ependymoma are tumors with high incidence in pediatric populations, with decreasing incidence in older age groups. This also applies to tumor sites, where tumors of the cerebellum are much more common in in pediatric ages than adult [11]. Brain and CNS tumors in general have much higher incidence relative to other types of cancers in children (0–14) and AYA (15–39) populations compared to adult populations [1, 12]. Additionally, pediatric brain tumor cases receive different treatment patterns cases due to higher risk of long term effects, particularly radiation and chemotherapy [11, 12]. Due to these variations in tumor incidence, behavior, and treatment across age groups, it is important to assess survival statistics for these groups separately.

A study of survival trends for primary central nervous system lymphomas in elderly individuals using CBTRUS and SEER data found that the median survival for all patients improved from 12.6 months to 26 months for patients diagnosed in the 1970s compared with patients diagnosed in 2010s [13]. The improvement in median survival supports the findings of the work presented here, in which median survival for lymphoma improved from 30 to 47 months in adults 40–64 years (from 2004 to 2007 to 2008–2012) and 7 to 13 months in older adults 65 + years (from 2004 to 2007 to 2013–2017) This increase in survival may be due to an increased use of combine modality treatment combined with strategies to minimize toxicity, as well as targeted immunotherapy though this remains to be determined [14–16]. For patients diagnosed with glioblastoma, significant advances in survival have come from the implementation of the Stupp protocol, concomitant treatment of radiation and temozolomide treatment, which saw significant increases in survival at two years and is now considered standard of care [17].

While this study provides the most comprehensive analysis of survival over time of brain and other CNS tumors in the US, it does not determine the root cause for this increase. A previous study, utilizing SEER data, evaluating survival over time in individuals diagnosed with glioblastoma found an improvement of 3 months from patients diagnosed in 2000–2001 to those diagnosed in 2005–2006 [18]. Using SEER registry data, Johnson and O’Neill analyzed the survival of individuals with glioblastoma prior to the implementation of the Stupp protocol (individuals diagnosed between 2000 and 2003) and after the implementation of the Stupp protocol (individuals diagnosed between 2005 and 2008) protocol and found a statistically significant improvement in survival with the implementation of this protocol [17]. There have been technological advances in imaging, which could have contributed to earlier and more accurate diagnosis. However, the impact of this remains to be determined and is beyond the scope of this study [19]. Decreasing the time from radiological diagnosis and an individual’s first surgery has been shown to have a positive impact on survival in individuals with low grade gliomas [20]. Unfortunately, this variable was not available in the dataset used for this analysis.

Another potential impact on changes in survival is the passage of the Affordable Care Act (ACA) in 2010 and the expansion of Medicaid, improving the survival outcomes in the later time period. The Medicaid expansion has been shown to be associated with greater use of cancer surgery by low-income individuals [21]. It may be interesting to hypothesize that the changes in survival observed here are due to increased diagnosis and treatment in low-income individuals. Studies have shown for cancers with early screening mechanisms that the passage of the ACA increased screening [22, 23]. Because brain tumors do not have screening mechanisms one could anticipate that the impact of the ACA on primary brain and other CNS tumors will be minimal. Indeed, a study by Moghavem et al. showed that implementation of the ACA did not have an impact on one year survival of individuals with glioblastoma [24]. Studies investigating the impact of the ACA on primary brain and other CNS tumors are warranted but are beyond the scope of the analysis presented here.

While survival changes over time in non-malignant brain tumor cases were small, there were some noted survival improvements in older adult (65+) patients. It is possible that improvements in detection, through changes in access, technology, and clinical practice are contributing factors to this change. Improvements in imaging (MRI, CT, angiography) seen in the last decade and its availability can result in advances in prognostication and surgical management, which may have impact survival. The impact of such imaging advances, particularly with non-malignant tumors warrants further investigation but is beyond the scope of this report [25, 26].

This study is not without limitations. The data here lacks a central pathology review for registry procedures, meaning that each case had been classified at the diagnosing institution with no central confirmation of histopathology at the state or national levels. In addition, data collection is based on passive rather than active follow up and some individuals, may be more prone to loss to follow up. Histopathologies have changed over time and these changes may impact survival patterns. In particular, the rules to diagnose grade 2 and grade 3 gliomas have undergone major changes over the time period analyzed here which may impact survival patterns. Due to the large scope of cases included in this paper, both microscopic and radiographic confirmation was used as selection criteria. Further studies, particularly focused on certain histopathologies, would be more robust by only including histologically confirmed tumors. NPCR survival data does not include detailed chemotherapy information, a strong prognostic factor, making it necessary to adjust the data based on surgery and radiation therapy [2] and care should be taken into account when interpreting results. Further, there is a lack of data regarding the extend of surgical resection of these tumors and should also be noted. There is evidence that supports molecular characterizations of brain tumors having distinct clinical characteristics. This information was not available for this study as cancer registry data did not have this data available for the years under study. Thus, the impact of the molecular characterization on survival could not be analyzed. Further studies to assess impact will be warranted once molecular information becomes more abundant (molecular information for brain and other CNS tumors was first collected for reporting year 2018). However, because this study utilized data covering 82% of all US individuals with reported malignant and non-malignant primary brain and other CNS tumors, this is the most current and comprehensive analysis of survival over time to date. This study evaluates survival over a 13-year period (2004–2017) with attention to age at diagnosis and most common histopathologies by age group, as this an established prognostic factor for cancer survival, with significant variation in types of brain tumor diagnoses across age group. However, further studies evaluating changes in primary brain tumors over time focused on other factors, such as race and ethnicity, are warranted.

## Conclusion

This study found individuals diagnosed in the later time periods (2008–2012, 2013–2017) had improved overall survival compared to individuals diagnosed earlier (2004–2007). Continued monitoring of changes in survival over time are important for adding to our understanding of advances in diagnosis and treatment of individuals with primary brain and other CNS tumors. Cancer registry-based statistics, such as these, provide critical information to physicians, clinical researchers, and other healthcare providers on disease burden and characteristics, which lead to improvements in patient care and prognosis.

## Electronic supplementary material

Below is the link to the electronic supplementary material.

Supplementary Material 1

## Data Availability

The datasets generated during and/or analyzed during the current study were obtained from the Centers for Disease Control and Prevention’s (CDC) NPCR survival database.
