# Supplementary material for: Changes in survival over time for primary brain and other CNS tumors in the United States, 2004–2017
Source: J Neurooncol. 2022 Oct 5;160(1):209–19. doi: 10.1007/s11060-022-04138-w (PMC9622549; doi:10.1007/s11060-022-04138-w)
Supplement: Supplementary file 1 — Supplementary Material 1 [file 11060_2022_4138_MOESM1_ESM.docx]

**Supplemental Table 1.** **Multivariable cox proportional hazard model results for primary malignant brain and other CNS tumors stratified by age at diagnosis adjusted for time period of diagnosis, sex, race/ethnicity, and treatment pattern.** (NPCR Survival Data: Data provided by CDC’s National Program of Cancer Registries SEER*Stat Database: NPCR Survival Analytic file, 2004–2017)

|  | 0-14 years | | | 15-39 years | | | 40-64 years | | | 65+ years | | |
| --- | --- | --- | --- | --- | --- | --- | --- | --- | --- | --- | --- | --- |
| Characteristic | HR^1^ | 95% CI^1^ | p-value | HR^1^ | 95% CI^1^ | p-value | HR^1^ | 95% CI^1^ | p-value | HR^1^ | 95% CI^1^ | p-value |
| **Time Period** |  |  |  |  |  |  |  |  |  |  |  |  |
| 2004-2007 | — | — |  | — | — |  | — | — |  | — | — |  |
| 2008-2012 | 0.92 | 0.87, 0.98 | 0.010 | 0.92 | 0.88, 0.96 | <0.001 | 0.99 | 0.97, 1.01 | 0.2 | 0.98 | 0.96, 1.00 | 0.043 |
| 2013-2017 | 0.91 | 0.85, 0.97 | 0.005 | 0.87 | 0.83, 0.93 | <0.001 | 0.97 | 0.95, 0.99 | 0.014 | 0.92 | 0.90, 0.94 | <0.001 |
| **Sex** |  |  |  |  |  |  |  |  |  |  |  |  |
| Female | — | — |  | — | — |  | — | — |  | — | — |  |
| Male | 0.92 | 0.88, 0.97 | 0.003 | 1.15 | 1.10, 1.19 | <0.001 | 1.15 | 1.13, 1.16 | <0.001 | 1.04 | 1.02, 1.06 | <0.001 |
| **Race/Ethnicity** |  |  |  |  |  |  |  |  |  |  |  |  |
| Non-Hispanic White | — | — |  | — | — |  | — | — |  | — | — |  |
| Non-Hispanic Black | 1.34 | 1.24, 1.44 | <0.001 | 1.22 | 1.14, 1.30 | <0.001 | 0.99 | 0.96, 1.03 | 0.7 | 0.79 | 0.76, 0.82 | <0.001 |
| Non-Hispanic Other | 1.16 | 1.03, 1.30 | 0.011 | 1.09 | 1.00, 1.19 | 0.047 | 0.75 | 0.71, 0.78 | <0.001 | 0.69 | 0.65, 0.73 | <0.001 |
| Hispanic (All Races) | 1.14 | 1.07, 1.22 | <0.001 | 0.97 | 0.92, 1.03 | 0.3 | 0.80 | 0.78, 0.82 | <0.001 | 0.80 | 0.77, 0.83 | <0.001 |
| **Treatment Pattern** |  |  |  |  |  |  |  |  |  |  |  |  |
| No Treatment | — | — |  | — | — |  | — | — |  | — | — |  |
| Radiation Only | 6.27 | 5.75, 6.83 | <0.001 | 2.61 | 2.41, 2.83 | <0.001 | 1.41 | 1.36, 1.46 | <0.001 | 0.64 | 0.62, 0.66 | <0.001 |
| Surgery and Radiation | 2.05 | 1.89, 2.23 | <0.001 | 1.93 | 1.80, 2.06 | <0.001 | 1.01 | 0.98, 1.04 | 0.7 | 0.40 | 0.40, 0.41 | <0.001 |
| Surgery Only | 1.88 | 1.73, 2.06 | <0.001 | 0.93 | 0.86, 1.01 | 0.069 | 0.83 | 0.81, 0.86 | <0.001 | 0.72 | 0.70, 0.74 | <0.001 |
| ^1^HR = Hazard Ratio, CI = Confidence Interval | | | | | | | | | | | | |

**Supplemental Table 2**. **Multivariable cox proportional hazard model results for primary non-malignant brain and other CNS tumors stratified by age at diagnosis adjusted for time period of diagnosis, sex, race/ethnicity, and treatment pattern.** (NPCR Survival Data: Data provided by CDC’s National Program of Cancer Registries SEER*Stat Database: NPCR Survival Analytic file, 2004–2017)

|  | 0-14 years | | | 15-39 years | | | 40-64 years | | | 65+ years | | |
| --- | --- | --- | --- | --- | --- | --- | --- | --- | --- | --- | --- | --- |
| Characteristic | HR^1^ | 95% CI^1^ | p-value | HR^1^ | 95% CI^1^ | p-value | HR^1^ | 95% CI^1^ | p-value | HR^1^ | 95% CI^1^ | p-value |
| **Time Period** |  |  |  |  |  |  |  |  |  |  |  |  |
| 2004-2007 | — | — |  | — | — |  | — | — |  | — | — |  |
| 2008-2012 | 0.95 | 0.79, 1.15 | 0.6 | 0.96 | 0.88, 1.05 | 0.4 | 0.97 | 0.94, 1.00 | 0.050 | 0.93 | 0.92, 0.95 | <0.001 |
| 2013-2017 | 0.78 | 0.61, 0.99 | 0.045 | 0.94 | 0.83, 1.06 | 0.3 | 0.97 | 0.93, 1.01 | 0.2 | 0.88 | 0.86, 0.89 | <0.001 |
| **Sex** |  |  |  |  |  |  |  |  |  |  |  |  |
| Female | — | — |  | — | — |  | — | — |  | — | — |  |
| Male | 0.95 | 0.81, 1.11 | 0.5 | 1.80 | 1.67, 1.95 | <0.001 | 1.49 | 1.46, 1.53 | <0.001 | 1.21 | 1.19, 1.22 | <0.001 |
| **Race/Ethnicity** |  |  |  |  |  |  |  |  |  |  |  |  |
| Non-Hispanic White | — | — |  | — | — |  | — | — |  | — | — |  |
| Non-Hispanic Black | 1.84 | 1.49, 2.27 | <0.001 | 1.53 | 1.39, 1.69 | <0.001 | 1.49 | 1.45, 1.55 | <0.001 | 1.12 | 1.10, 1.14 | <0.001 |
| Non-Hispanic Other | 0.91 | 0.58, 1.44 | 0.7 | 0.75 | 0.61, 0.91 | 0.004 | 0.76 | 0.71, 0.82 | <0.001 | 0.72 | 0.69, 0.75 | <0.001 |
| Hispanic (All Races) | 1.26 | 1.03, 1.54 | 0.024 | 0.88 | 0.79, 0.98 | 0.023 | 0.87 | 0.83, 0.91 | <0.001 | 0.82 | 0.80, 0.84 | <0.001 |
| **Treatment Pattern** |  |  |  |  |  |  |  |  |  |  |  |  |
| No Treatment | — | — |  | — | — |  | — | — |  | — | — |  |
| Radiation Only | 4.58 | 3.13, 6.72 | <0.001 | 1.33 | 1.08, 1.64 | 0.007 | 0.59 | 0.56, 0.63 | <0.001 | 0.57 | 0.55, 0.59 | <0.001 |
| Surgery and Radiation | 2.72 | 2.00, 3.71 | <0.001 | 2.30 | 1.98, 2.67 | <0.001 | 0.92 | 0.86, 0.99 | 0.018 | 0.60 | 0.57, 0.64 | <0.001 |
| Surgery Only | 1.01 | 0.84, 1.21 | >0.9 | 1.08 | 1.00, 1.18 | 0.056 | 0.64 | 0.62, 0.66 | <0.001 | 0.53 | 0.52, 0.54 | <0.001 |
| ^1^HR = Hazard Ratio, CI = Confidence Interval | | | | | | | | | | | | |
